# Chest Pain and Costochondritis Associated with Vitamin D Deficiency: A Report of Two Cases

**DOI:** 10.1155/2012/375730

**Published:** 2012-06-12

**Authors:** Robert C. Oh, Jeremy D. Johnson

**Affiliations:** Department of Family Medicine, Tripler Army Medical Center, Honolulu, HI 96859, USA

## Abstract

Vitamin D is integral for bone health, and severe deficiency can cause rickets in children and osteomalacia in adults. Although osteomalacia can cause severe generalized bone pain, there are only a few case reports of chest pain associated with vitamin D deficiency. We describe 2 patients with chest pain that were initially worked up for cardiac etiologies but were eventually diagnosed with costochondritis and vitamin D deficiency. Vitamin D deficiency is known to cause hypertrophic costochondral junctions in children (“rachitic rosaries”) and sternal pain with adults diagnosed with osteomalacia. We propose that vitamin D deficiency may be related to the chest pain associated with costochondritis. In patients diagnosed with costochondritis, physicians should consider testing and treating for vitamin D deficiency.

## 1. Introduction

Chest pain is a leading cause of ambulatory visits and accounts for over 6 million emergency room visits in the United States [1]. After serious cardiopulmonary conditions are considered, musculoskeletal causes of chest pain, including costochondritis, are commonly attributed to the final diagnosis [2, 3]. Costochondritis is not clearly understood, but may be related to inflammation of the costochondral junctions associated with illness, coughing, or trauma [3]. Although there are a few reports of osteomalacia and vitamin D deficiency associated with chest pain [4–6], we are not aware of any literature reports of costochondritis associated with vitamin D deficiency. This paper describes two cases of patients with chest pain, eventually diagnosed with costochondritis and vitamin D deficiency.

## 2. Cases

**Case 1**

A 35-year-old white female presented to the family medicine clinic in Hawaii with complaints of chronic chest pain for the last 3 years. She recently moved from Northern Virginia to Hawaii. Chart review was notable for a negative cardiac workup, including a treadmill stress test, and echocardiogram. Over the last 3 years, the diagnoses of her chest pain included anxiety, esophageal reflux, and costochondritis. In Hawaii, her exam was remarkable only for tenderness to palpation over the left and right costochondral junctions. Since she reported very little milk intake or sun exposure, a serum 25-OH Vitamin D (25-OHD) level was obtained and returned at 42 nmol/L (17 ng/mL), consistent with deficiency. She was started on Vitamin D, 1000 international units (IU) daily. On 3-month followup, her repeat serum 25-OHD was 72 nmol/L (29 ng/mL) and she had complete resolution of her chest pain.

**Case 2**

A 42-year-old Asian female with historcarotid Doppley of hypertension and hyperlipidemia presented to a Hawaii emergency department with complaints of substernal chest pain. She was admitted to the hospital for a rule-out myocardial infarction protocol. In the hospital, she was found to have marked tenderness along the left costochondral junction. She did not routinely drink milk and despite living in Hawaii, she reported little sun exposure due to her work hours. After ruling out for myocardial infarction, she was discharged with followup for a treadmill stress test, which was normal. Prior to her discharge, a serum 25-OHD level was drawn to rule out vitamin D deficiency. On followup, her vitamin D level returned 27 nmol/L (11 ng/mL) and she continued to report chest pains. She was started on oral Vitamin D_2_ 50,000 IU once a week for 8 weeks and maintained on 1,000 IU a day thereafter. Repeat 25-OHD level 2 months later was 82 nmol/L (33 ng/mL). Her chest pain resolved with treatment.

## 3. Discussion

In this paper, both patients had extensive workups done with concern for cardiac disorders but were eventually diagnosed with costochondritis and vitamin D deficiency. We hypothesize that these patients' costochondritis may have been related to vitamin D deficiency. Vitamin D is integral for bone health, and serum 25-OH vitamin D (25-OHD) is predictive for body stores of vitamin D [7–9]. While there is controversy over what defines deficient or optimal serum levels of 25-OHD, levels less than 50 nmol/L (20 ng/mL) lead to increase in bone turnover markers and increase in parathyroid hormone (PTH) [10]. Another study found that defective bone mineralization was evident in patients with serum 25-OHD less than 75 nmol/L (30 ng/mL) but none above that threshold [11]. The recent Institute of Medicine report found evidence that a serum 25-OHD level above 50 nmol/L (20 ng/mL) is generally sufficient for bone health for 97.5% of the population. Hence, a 25-OHD level less than 50 nmol/L (20 ng/mL) is generally considered consistent with vitamin D deficiency [7, 8]. Severe vitamin D deficiency, defined as a 25-OHD less than 25 nmol/L (10 ng/mL), can cause rickets in children and osteomalacia in adults [12]. Rickets classically causes “bow legs” or “knock-knees” due to disordered growth along weight-bearing bone. “Rachitic rosaries” describes hypertrophied costochondral junctions in relation to defective mineralization [13, 14]. Osteomalacia, the adult version of rickets, can cause diffuse bone pain. Tenderness to the anterior tibia, sternum, and costochondral joints can indicate osteomalacia and vitamin D deficiency [14, 15]. Although bone biopsy is the gold standard for osteomalacia, it is not generally practiced. Bone pain and vitamin D levels less than 25 nmol/L (10 ng/mL) are often sufficient for the clinical diagnosis of osteomalacia. 

Although both patients did not have had levels less than 25 nmol/L (10 ng/mL) to suggest osteomalacia, we theorize that milder forms of vitamin D deficiency can cause a spectrum of pain along the sternum and costochondral junctions similar to patients with rickets and osteomalacia. Costochondritis continues to be a poorly defined entity but may represent a milder, earlier form of osteomalacia associated with higher serum 25-OHD levels. Importantly, osteomalacia and vitamin D deficiency may not be considered when a patient presents with complaints consistent with costochondritis, as testing for vitamin D deficiency in these patients has not been reported, nor is it routine. 

A review of the literature revealed no reported cases of costochondritis associated with vitamin D deficiency, and only a few case reports of chest pain associated with vitamin D deficiency. One case described chronic chest and leg pain of 2 years duration in a 34-year-old female [4]. After two years of conservative treatment with anti-inflammatory medications, she was eventually diagnosed with osteomalacia associated with aluminum-containing antacid use. Aluminum containing antacids may cause osteomalacia by binding to phosphate, causing a negative phosphate balance and eventual disordered bone growth. However, there was no serum 25-OH vitamin D (25-OHD) level measured to support the diagnosis of osteomalacia. She improved with vitamin D supplementation and decreased antacid use. Another case reported a 37-year-old Indian living in Germany who was subsequently diagnosed with vitamin D deficiency and osteomalacia. Chest pain was the only presenting symptom. His 25-OHD level was undetectable. He was started on high-dose vitamin D and then maintenance vitamin D with resolution of his chest pain [5]. Lastly, a 21-year-old Turkish female living in Germany presented with left-sided chest pain with unremarkable electrocardiogram, troponin and d-dimer [6]. Her 25-OHD level was 9 nmol/L (4 ng/mL), and she was diagnosed with osteomalacia causing her chest pain. Treatment with vitamin D, calcium, and calcitonin resolved her symptoms in two months. These case reports are suggestive of osteomalacia and severe vitamin D deficiency as the cause of chest pains. It is possible that the spectrum of bone pain, including costochondritis, may be correlated to the degree of vitamin D deficiency.

In our paper, both patients were at significant risk for vitamin D deficiency despite living in an area with abundant sunshine. Vitamin D can only be obtained through ultraviolet light exposure, vitamin-D-rich foods, and supplementation. Both patients reported little to no milk intake, no other vitamin supplementation, and little sun exposure, putting them at high risk for deficiency. The IOM recently increased the recommended daily allowance (RDA) of vitamin D for adults from 400 IU to a minimum of 600 IU in order to maintain 25-OHD levels above 50 nmol/L (20 ng/mL) for 97.5% of the population [7]. However, other expert recommendations have suggested much higher levels of vitamin D, ranging from 400 to 2000 IU daily to maintain adequate serum 25-OHD; depending on risk factors present [8, 9]. Sun exposure can provide the most efficient means of maintaining vitamin D stores; however, dark-skinned individuals absorb less ultraviolet radiation from sunlight and thus convert less 7-dehydrocholesterol into vitamin D. People living at higher latitudes are at increased risk for deficiency, but studies show that even in areas with adequate sunshine, vitamin D deficiency can be highly prevalent [16, 17].

Our paper has clear limitations. With prevalence of vitamin D deficiency reported to be up to 36% in young adults [12], costochondritis may be merely an unrelated association. However, with treatment of deficiency and normalization of their serum vitamin D, both patients' costochondritis improved, further supporting vitamin D deficiency as a potential cause of their costochondritis. The improvement of symptoms may also be attributed to self-limited costochondritis rather than the vitamin D supplementation. However, it is notable that once adequate treatment was initiated, no further cases of chest pains have been reported—including the patient who had 3 years of chronic chest pain. This further strengthens our hypothesis that vitamin D deficiency may be a cause of costochondritis. We recommend further prospective studies, specifically in primary care, to help elucidate the association of vitamin D deficiency and costochondritis.

## 4. Conclusion

In patients with costochondritis, or bony sternal pain, physicians should consider vitamin D deficiency and osteomalacia and elicit any risk factors for deficiency. Patients with costochondritis who are at risk for vitamin D deficiency should be tested with a serum 25-OHD level and treated if found to be vitamin D deficient. Studies looking further at the association of costochondritis and vitamin D deficiency are warranted.
